# Insights into a long life without cancer: The case of the bowhead whale

**DOI:** 10.1002/1878-0261.70250

**Published:** 2026-04-10

**Authors:** Inés Paniagua, Johanna A. Joyce

**Affiliations:** ^1^ Department of Fundamental Oncology University of Lausanne Switzerland; ^2^ Ludwig Institute for Cancer Research University of Lausanne Switzerland; ^3^ Agora Cancer Research Centre Lausanne Switzerland

**Keywords:** cancer resistance, CIRBP, DNA repair, genome stability, longevity, Peto's paradox

## Abstract

Long‐lived and large‐bodied organisms face an inherent challenge: The more cells they contain and the longer they live, the greater the cumulative risk of acquiring mutations that can drive cancer. Yet, paradoxically, cancer incidence does not always scale with size or lifespan—a phenomenon known as Peto's paradox. This observation implies that some species have evolved highly effective anticancer mechanisms that preserve cellular and tissue integrity over long lifespans. Whales, which combine extreme longevity with vast numbers of cells, exemplify this paradox. In this commentary, we discuss a recent study showing that a key contributor to bowhead whales' exceptional lifespan and cancer resistance is their superior genome maintenance capacity. We further discuss DNA repair as a determinant of longevity in other long‐lived species and explore how these naturally occurring mechanisms could be harnessed to improve genome integrity, reduce cancer risk, and promote healthy aging in humans.

## Graphical abstract

Long‐lived, large‐bodied organisms have evolved powerful anticancer mechanisms that preserve cellular and tissue integrity across extended lifespans. A recent study by Firsanov *et al.* shows that greater genome stability is a key factor underlying the remarkable longevity and cancer resistance of one such species, the bowhead whale. Building on these findings, we discuss enhanced genome stability and DNA repair as determinants of longevity in other long‐lived species and highlight how these naturally occurring mechanisms could be harnessed to improve genome integrity, reduce cancer risk, and promote healthy aging in humans.

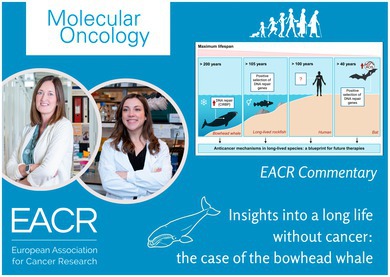

**Abbreviations**
DSB: DNA Double‐Stranded Breaks
HR: Homologous Recombination
NHEJ: Non‐Homologous End‐Joining

Across the animal kingdom, cancer risk should, in principle, scale with increasing body size and lifespan. Larger animals contain vastly more cells, and longer lives permit many rounds of cell division, both of which increase the probability of accumulating mutations and undergoing malignant transformation. However, this expectation does not always hold in nature. Some of the largest and longest‐lived animals, such as whales and elephants, exhibit a remarkably low cancer incidence. This incongruency, known as Peto's paradox [1], suggests that evolutionary pressures have endowed these animals with unusually potent anticancer mechanisms to counteract the mutational burden associated with large bodies and extended lifespans. Indeed, studies in elephants revealed that these large animals have evolved multiple copies of the tumor suppressor *TP53* gene, which is associated with an increased apoptotic response that allows the prompt elimination of damaged cells before they become precancerous [2, 3]. Interestingly, sequencing and analysis of several whale genomes [4, 5], including the bowhead whale [6]—known to live for over 200 years—did not reveal the *TP53* duplications seen in elephants, suggesting that whales rely on alternative, previously uncharacterized, anticancer strategies. Recently, a landmark study published in *Nature* [7] offered key insights into the molecular and cellular pathways that underpin the extraordinary longevity and cancer resistance of these marine giants.

## 1 Decoding whales' exceptional longevity

In this seminal investigation, Firsanov *et al*. [7] first obtained primary fibroblasts from skin and lung tissues of adult bowhead whales harvested by Iñupiaq Inuit hunters in northern Alaska as part of their long‐standing subsistence traditions. Given the whale's exceptional longevity, the authors hypothesized that whale cells would be intrinsically resistant to malignant transformation and therefore require substantially more oncogenic mutations to induce cancer. Surprisingly, they observed the opposite: whale fibroblasts underwent malignant transformation with fewer mutational hits than required for human fibroblasts. This unexpected result suggested that cancer resistance in bowhead whales might not come from an increased tolerance to oncogenic mutations, but rather from mechanisms that prevent or limit their accumulation altogether. Consistent with this idea, whole‐genome sequencing revealed a strikingly low mutational burden in whale tumors, with fewer somatic single‐nucleotide variants, insertions and deletions, and large‐scale structural alterations than those observed in human or mouse tumors. One potential explanation for this finding is a superior ability to maintain genome integrity, which prompted the authors to investigate DNA repair capacity across multiple mammalian species.

**Fig. 1:**
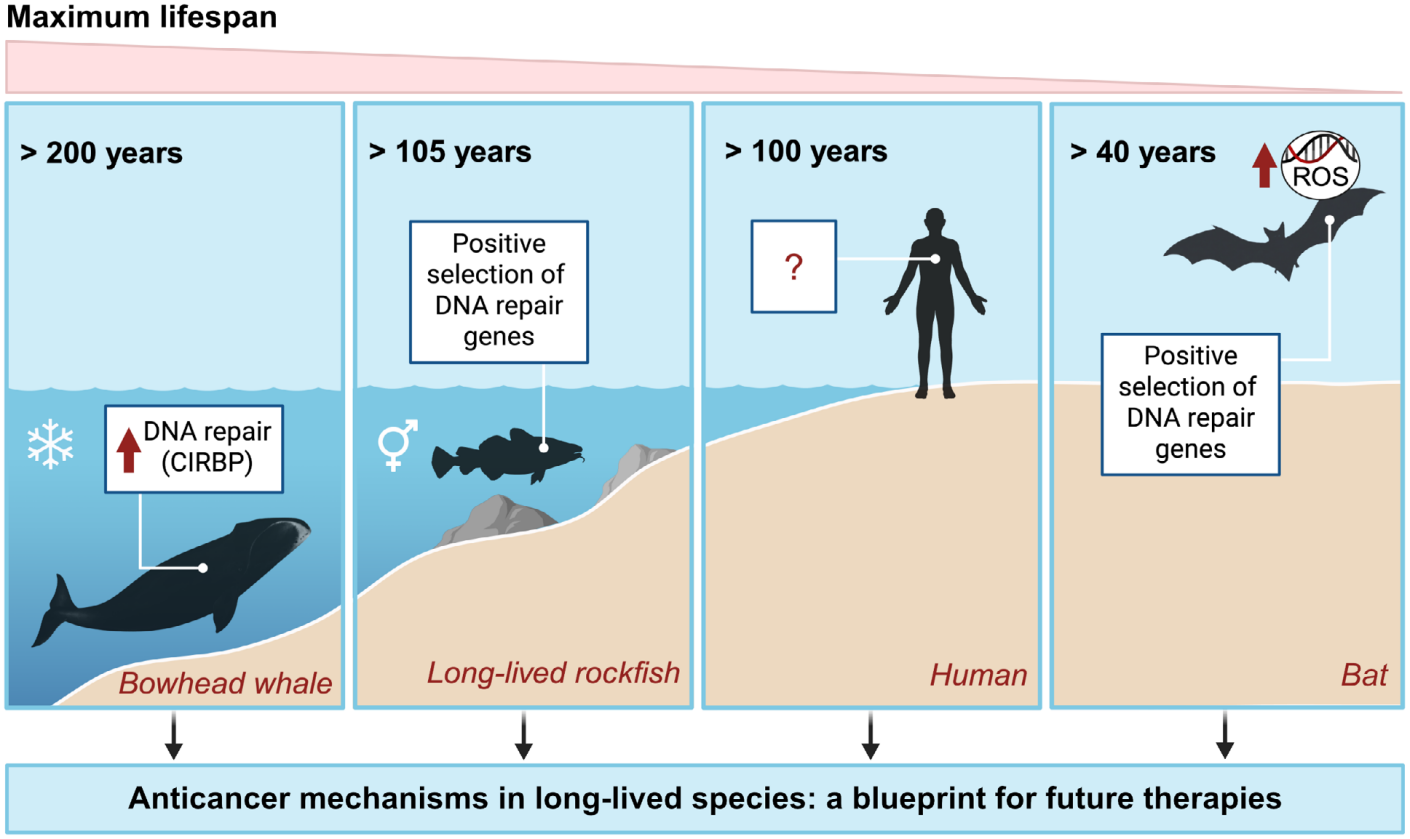
Evolutionary strategies for cancer resistance in long‐lived species. Long‐lived species have evolved diverse mechanisms to maintain genome integrity under their specific ecological and physiological pressures. Bowhead whales, exposed to extreme cold, exhibit enhanced DNA repair partly mediated by the cold‐inducible protein CIRBP; long‐lived rockfish, whose fecundity increases with age, show positive selection of key DNA repair genes; bats, challenged by high metabolic rates and reactive oxygen species generated during flight, also display positive selection of DNA repair genes to preserve genome stability. In human centenarians, such adaptations remain largely uncharacterized. Understanding the mechanisms responsible for cancer resistance in long‐lived species will have a transformative effect on developing interventions to improve human health and longevity.

Indeed, whale fibroblasts exhibited more efficient and accurate repair of DNA double‐stranded breaks (DSB), with significantly higher activity of both non‐homologous end‐joining (NHEJ) and homologous recombination (HR), as well as enhanced mismatch repair. To investigate the underlying mechanisms, the authors performed comparative proteomic and transcriptomic analyses and identified the cold‐inducible RNA‐binding protein CIRBP as the most prominently upregulated DNA repair factor in bowhead whales (Fig. 1). Overexpression of whale CIRBP in human cells enhanced both NHEJ and HR efficiency, whereas CIRBP depletion reduced the efficiency of these repair pathways and increased the prevalence of DNA deletions. Although the precise mechanisms remain to be fully elucidated, the data are consistent with a model in which CIRBP is recruited to DNA DSBs, where it promotes the recruitment and/or activation of DNA damage response factors and transient DNA end protection. Strikingly, expression of either human or bowhead whale CIRBP in *Drosophila* extended lifespan and increased resistance to mutation‐inducing radiation [7], underscoring its conserved and potent role in promoting genome integrity and organismal longevity.

## 2 Enhanced DNA repair as a longevity determinant

This study adds to a growing body of literature demonstrating that exceptional longevity and cancer resistance across diverse taxa are tightly linked to superior genome maintenance capacity, with DNA repair emerging as a key determinant of lifespan. For instance, a comparative study of 18 rodent species with vastly different lifespans found that longer‐lived rodents have more efficient DSB repair, driven in part by a more potent version of the genome maintenance protein SIRT6 [8]. In bats, which are both long‐lived and cancer‐resistant, positively selected genes are enriched for core DNA damage signaling and repair factors, including ATM, RAD50, PRKDC, and XRCC5 [9]. Similarly, long‐lived rockfish species (> 105 years) show significant enrichment of positively selected genes involved in DSB repair pathways (Fig. 1) [10].

But why are DNA repair genes repeatedly subject to positive selection in long‐lived species? One plausible explanation is that enhanced genome maintenance represents an adaptive response to the ecological and physiological stresses that act over a long lifespan. In bats, the high metabolic demands of flight generate elevated levels of reactive oxygen species, which can induce oxidative DNA damage, increasing the need for more efficient DNA repair [9]. In long‐lived rockfish, survival to old age confers clear reproductive benefits, as fecundity increases with age, thereby favoring the selection of DNA repair genes that protect against age‐associated DNA damage [10]. More broadly, environmental factors such as UV radiation, extreme temperatures, and pathogen pressure may further shape selection on specific DNA repair pathways. In this context, it is notable that whales exhibit high levels of the cold‐inducible protein CIRBP [7], which may represent an adaptation to life in ice‐cold waters, helping to preserve tissue integrity under cold‐induced stress. Notably, Firsanov *et al*. observed that human primary fibroblasts similarly increase CIRBP expression and NHEJ frequency in response to mild hypothermia (33 °C) [7]. Together, these intriguing findings suggest that this stress‐responsive genome maintenance program is evolutionarily conserved across species.

## 3 Harnessing natural anticancer mechanisms for therapeutic use

Natural anticancer mechanisms provide a compelling blueprint for improving human health. If we can identify the DNA repair pathways that contribute to cancer resistance in long‐lived species, and functionally validate them *in vivo*, we may uncover new strategies for preventing or treating cancer in humans (Fig. 1). For example, overexpression of the cold‐inducible protein CIRBP in mice could potentially enhance protection against DNA damage, promote cancer resistance, or even extend lifespan. Such findings could guide the development of pharmacological strategies that mimic the effects of CIRBP overexpression or other genome‐protective adaptations observed in long‐lived organisms.

Beyond genetic manipulation, these insights also raise the possibility of physiological interventions. The reported increase in CIRBP expression and DNA repair activity following mild hypothermia [7] raises the provocative possibility that controlled cold exposure—such as cold plunges or cryotherapy—could be harnessed to transiently or sustainably boost genome maintenance pathways, as suggested by the authors. Importantly, if such cold‐induced genome‐protective programs extend to stem cell compartments, the implications could be substantial. Stem cells rely on robust DNA repair to preserve long‐term function yet progressively accumulate damage over time. Enhancing DNA repair in hematopoietic stem cells through CIRBP overexpression or related cold‐responsive pathways could therefore reduce mutational burden, maintain stem cell fitness, and potentially extend organismal lifespan. In oncology, this strategy could be used to generate blood stem cells with increased resistance to chemotherapy or radiation, thereby limiting treatment‐associated toxicity. Despite the long‐standing association of cold exposure with health benefits, such as reduced inflammation and improved tissue recovery, its impact on DNA repair and genome stability remains largely unexplored and represents an intriguing area of future investigation.

## 4 Conclusion

Ultimately, as longevity and cancer resistance—within and across species—are likely driven by multiple interconnected mechanisms rather than a single dominant pathway, the study of long‐lived organisms is expected to continue yielding important insights into fundamental principles of genome maintenance. Viewed in this light, Peto's paradox is not a problem to solve, but rather a framework for understanding how evolution has repeatedly arrived at effective and diverse solutions to the challenge of maintaining genomic integrity over extended lifespans.

Many of these solutions remain only partially charted, embedded in the biology of species that have evolved under extreme ecological and physiological constraints. As Herman Melville reflected in *Moby‐Dick* [11], his novel centered on humanity's encounter with the whale as a vast, powerful, and poorly understood force of nature: “*It is not down in any map; true places never are*.” Deciphering these naturally evolved strategies may not only illuminate the biology of aging and cancer resistance but also inspire new approaches to cancer prevention and healthy aging in humans.

## Conflict of interest

The authors declare no conflict of interest.

## Author contributions

IP and JAJ wrote the manuscript. IP prepared the figure.
